# A Survey of Imidacloprid Levels in Water Sources Potentially Frequented by Honeybees (*Apis mellifera*) in the Eastern USA

**DOI:** 10.1007/s11270-014-2127-2

**Published:** 2014-10-19

**Authors:** J. D. Johnson, J. S. Pettis

**Affiliations:** Bee Research Laboratory, USDA Agricultural Research Service, BARC-East, Beltsville, MD 20705 USA

**Keywords:** Pesticide, Surface water, Honeybees, Imidacloprid, Pollution

## Abstract

Imidacloprid, a water-soluble neonicotinoid pesticide used globally in many applications, has been the subject of numerous studies (1) to determine its sublethal effects (5–100 ppb, LD_50_ ∼200 ppb) on honeybees. This study was undertaken to determine, by ELISA assay, the presence of imidacloprid in water sources potentially frequented by honeybees in urban, suburban, and rural environments across the state of Maryland. Eighteen sites (six samples/site) were chosen which spanned diverse habitats including golf courses, nursery, livestock and crop farms, residential neighborhoods, and cityscapes. Hives were present either at or within 0.5 miles of each site. Imidacloprid was quantifiable in 8 % of the samples at sublethal levels (7–131 ppb). They were not clustered at any one type of site. Results for 13 % of the samples were at the threshold of detection; all others were below the detection limit of the assay (<0.2 ppb).

This study was undertaken to examine contamination levels of imidacloprid (IMI), a water-soluble neonicotinoid insecticide, in still or slow-moving water sources of the sort often frequented by honeybees, *Apis mellifera*. Honeybees frequent open water to transport water into the hive for consumption, cooling of the hive (Kuhnholz and Seeley 1997), dilution of honey for brood use, and humidity maintenance for brood rearing (Gould and Gould 1995). If water sources frequented by honeybees carry low levels of pesticides, the contamination, by contact or by ingestion, may adversely affect their health.

IMI is ubiquitously used in many applications and has been found in the environment since its introduction in 1991 by Bayer CropScience (Jeschke et al. 2011). The pesticide moves systemically by xylem transport through treated plants mostly to leaves and, to a lesser extent, flowers (Sur and Stork 2003; Diaz and McLeod 2005; Byrne et al. 2010; Romeh 2010). It has been detected in soil in years following application (Scholz and M. Spiteller 1992; Miles Inc 1993; Rouchaud et al. 1996; Cox et al. 1997, 1998; Bonmatin et al. 2000; Krupke et al. 2012), guttation water (Girolami et al. 2009; Tapparo et al. 2011), and leaf litter (Kreutzweiser et al. 2009). Early water surveys reported occasional detections of IMI in water systems: a surface water survey (38 sites) detected one sample at 1.0 ppb in Florida (Pfeuffer and F. Matson 2001) and a surface water survey (47 sites) detected two samples at 0.07 and 0.2 ppb in New York (Phillips and R.W. Bode 2002). More recently, in the Netherlands, van Dijk (2010) reports that the MTR (maximum allowable risk level at which the species in an ecosystem are safe from effects caused by the substance) limit of 0.013 μg/l IMI was exceeded by 1,345 out of 4,852 samples and Starner and Goh (2012) report that the US Environmental Protection Agency’s chronic invertebrate aquatic life benchmark limit of 1.05 μg/l IMI (EPA 2008) was exceeded by 14 samples (19 % of total samples) in California, USA. Blacquiere et al. (2012) provide a review of sublethal effects of imidacloprid on honeybees, and the meta-analysis studies conducted by Cresswell (2011) and Halm et al. (2006) provide insightful review as well. The LD_50_ reported for honeybees ranges from 4 to 104 ng/honeybee or ∼25 to 612 ppb (Nauen et al. 2001; Schmuck et al. 2001; Decourtye et al. 2003; Iwasa et al. 2004; Suchail et al. 2001, 2004), but Mullin et al. (2010) report 280 ppb IMI or ∼48 ng/adult bee as an average LD_50_ from the literature for the body burden of this pollinator.

The intent of the study was to determine, by ELISA assay, the amount of imidacloprid in water sources that are likely to be visited by honeybees. In rural areas, honeybee water sources were anticipated to include low puddles in fields, small streams, and wetlands, and in residential and urban areas, sources were anticipated to include storm management ponds, street drain puddles, koi ponds, fountains, and potted plant holders. Eighteen distinct sites spanning Maryland’s agricultural Eastern Shore to the Pennsylvania line and including suburban/urban areas in or near Baltimore, Annapolis, and Washington, DC, Fig. 1a, were chosen which surveyed diverse habitats including livestock and crop farms, residential neighborhoods, and cityscapes. Hives were present within 0.5 miles of each site.

**Fig. 1:**
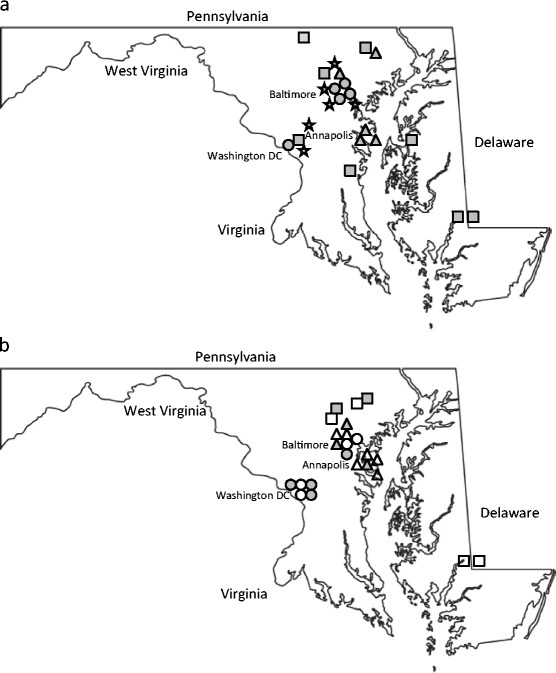
**a** Site locations. Symbols designate site descriptions: *circle* = urban, *triangle* = suburban, *square* = rural, *star* = control. **b** Map of all samples positive for IMI in Maryland. Symbols designate site descriptions: *circle* = urban, *triangle* = suburban, *square* = rural. *Darkened symbols* represent samples with quantifiable amounts of IMI. *Open symbols* represent samples for which IMI was at the threshold of detection. Quantifiable samples represent 8 % of the total samples collected. Threshold values represent 13 % of the total samples collected

Water samples (∼10 ml) were collected in 15-ml new plastic opaque cylindrical vials with screw top lids from potential honeybee surface water sources. One sample each was taken from three separate household taps and from deionized distilled water tanks in three separate research labs to serve as six controls. Vials with water were held on ice in the field, shipped within 3 days to the Animal and Plant Health Inspection Service lab at Otis ANGB, Massachusetts, and stored in a dark refrigerator at −20 °C until analysis 14 weeks later. The ELISA assay (EP 006 Imidacloprid QuantiPlate Kits, EnviroLogix, Portland, ME) consisted of a competition of horseradish peroxidase-labeled IMI with free IMI for a limited number of antibody sites, causing a color change that lightens with a higher concentration of IMI. This assay is specific for water samples with an assay range of 0.2 to 6 ppb IMI (limit of detection (LOD) = 0.07 ppb and limit of quantitation (LOQ) = 0.2–0.3 ppb). The assay is temperature sensitive and precautions were taken to respect temperature considerations. Standard concentrations of IMI were supplied with the assay kit and were applied to a row of wells on the same test plate as the unknown samples to serve as controls. The metabolites olefin, des nitro, and urea and two other neonicotinoids are detectable at levels similar to IMI. Clothianidin and thiamethoxam, two congeners of IMI, are detectable by this test but have LODs that are ∼100× higher than the LOD for IMI. Several other (non-neonicotinoids) pesticides (26 listed) have no cross reactivity up to 1,000 ppb (Envirologix 2010).

Table 1 provides descriptions of the samples and the ELISA results from June 2010. Positive quantifiable results of the ELISA assay (*n*_total_ = 108) ranged from 7 to 131 ppb IMI in nine samples equally distributed in urban, suburban, and rural settings. Fourteen of the samples were not quantifiable by ELISA (0.2 < *x* < 0.3 ppb IMI). Using a value of 0.25 ppb for each of the threshold values, the average IMI concentration for all 108 samples was 2.45 ppb by ELISA. The average for all 23 positive ELISA-analyzed samples (quantifiable and threshold, see Fig. 1b for locations) was 11.5 ppb IMI. In total, 21 % of the samples surveyed for IMI by ELISA were at or above the threshold of detection for the ELISA assay.

**Table 1:** IMI results (ppb) determined by ELISA. Descriptions are provided for water sources sampled at each site. Only numeric or threshold (0.2–0.3 ppb IMI) results followed by the sample description are reported in the positive sample column

Setting	Site description	Negative samples	Positive samples
Urban	Center city	1 puddle, 2 fountains, bird bath, car wash	22 ppb puddle
Urban	Nursery	1 puddle	131 and 7 ppb puddles, 27 ppb water tank, 2^a^ puddles
Urban	Golf course	4 rivulets, puddle, culvert	
Urban	City townhouses	4 fountains, statue with standing water, bird bath	
Urban	Close free-standing houses	2 puddles, drainpipe, fish pond	2^a^ small pools
Suburban	Residential	6 rivulets	
Suburban	Residential	Storm management pond, fishpond, fountain, water kettle	12 ppb puddle,
			1^a^ puddle
Suburban	Residential	2 drainage ditches, 1 puddle, fishpond	2^a^ drainage ditch, puddle
Suburban	Golf course	1 rivulet, 1 puddle	10 ppb rivulet
			8 ppb pond
			2^a^ rivulets
Suburban	Nature center	2 ponds, marsh, rain barrel, sapling starter tray	1^a^ pond
Rural	Crop/livestock farm	2 ponds, 2 rivulets, puddle, drainpipe	
Rural	Crop farm	5 irrigation pipes	1^a^ irrigation pipe
Rural	Golf course	3 ponds, 1 rivulet	25 ppb pond
			1^a^ pond
Rural	Farm	2 rivulets, 2 ponds, forest wetland	1^a^ hog farm runoff
Rural	Orchard	3 springs, 3 rivulets, pond (extra sample)	
Rural	Cattle farm	3 springs, 1 stream	19 ppb stream
			1^a^ pond
Rural	Sod farm	4 ditches, rivulet, wetland	
Rural	Crop farm	3 rivulets, 2 ponds, lowland	
Urban/suburban controls	Household or research lab	3 samples household tap water, 3 samples deionized distilled lab water	

^a^Result is LOD < [*x*] < LOQ

The ELISA test is designed to be most sensitive to IMI. IMI degrades in water to IMI urea, 6-chloronicotinic aldehyde, 6-chloro-*N*-methylnicotinacidamide, and 6-chloro-3-pyridyl-methylethylenediamine (Fossen 2006). IMI urea behaves similarly but slightly less sensitively in the ELISA test, and any contributions to a decrease in absorbance would be an indirect reflection of IMI concentration since IMI urea is a breakdown product of IMI in water. The other three hydrolysis metabolites are not quantified in the ELISA assay. Other IMI metabolites and four substances in the neonicotinoid class, thiacloprid, acetamiprid, clothianidin, and thiamethoxam, are reported to react but at a lower sensitivity (Envirologix 2010). Imidacloprid, the most likely contributor to the ELISA absorbance changes, was concluded to be present in water sources taken from rural to urban settings but was found most consistently in golf course and nursery sites. One sample, the rural cattle farm concentration of 19 ppb in a stream (Table 1), was a surprisingly high result, considering that no golf courses were apparent and the land was not heavily farmed. The steepness of the creek walls may have diminished seepage of water into the soil and subsequent slowing of IMI movement. If IMI had been present or applied upstream, it could have been concentrated into the creek by the topography. IMI is slow to degrade under conditions of neutral pH and dark storage (Sarkar et al. 1999; Wamhoff and Schneider 1999). The results from this study suggest that IMI is present in all environments (urban to rural).

Assessing the exposure levels of IMI on honeybee health is complicated. A sample such as the nursery puddle sample containing 131 ppb may be high enough to kill a small percentage of a nearby population of bees, but IMI concentrations in honeybee water sources seem to exist mostly at low sublethal doses which should pose less risk to the health of the colony. Changes in water movement and volume such as evaporation increasing a puddle concentration or rainfall diluting a concentration would make quantification of a water-soluble pesticide a time- or weather-dependent event. Hives near golf courses and nurseries where IMI is likely to be regularly applied might present the highest risk of exposure. This risk could be mitigated by the presence of alternate pesticide-free water sources provided naturally or by an apiarist.

## References

[CR1] Blacquiere T, Smagghe G, van Gestel CAM, Mommaerts V (2012). Neonicotinoids in bees: a review on concentrations, side effects and risk management. Ecotoxicology.

[CR2] Bonmatin, J.-M., Bengsch, E. R., Moineau, I., Lecoublet, S., Colin, M. E., & Fleche, C. (2000). Effets des produits phytosanitaires sur les abeilles. Programmes 1999 et 2000. Rapport de resultants no.3 au. Paris Ministere de l’Agriculture et de la Peche. 32 pp.

[CR3] Byrne FJ, Humeres EC, Urena AA, Hoddle MS, Morse JG (2010). Field evaluation of systemic imidacloprid for the management of avocado thrips and avocado lace bug in California avocado groves. Pest Management Science.

[CR4] Cox L, Koskinen W, Yen P (1997). Sorption–desorption of imidacloprid and its metabolites in soils. Journal of Agricultural and Food Chemistry.

[CR5] Cox L, Koskinen W, Celis R, Yen P, Hermosin M, Cornejo J (1998). Sorption of imidacloprid on soil clay mineral and organic components. Soil Science Society of America Journal.

[CR6] Cresswell JE (2011). A meta-analysis of experiments testing the effects of a neonicotinoid insecticide (imidacloprid) on honey bees. Ecotoxicology.

[CR7] Decourtye A, Lacassie E, Pham-Delègue MH (2003). Learning performances of honeybees (*Apis mellifera* L.) are differentially affected by imidacloprid according to the season. Pest Management Science.

[CR8] Diaz FJ, McLeod P (2005). Movement, toxicity, and persistence of imidacloprid in seedling Tabasco pepper infested with *Myzus persicae* (Hemiptera: Aphididae). Journal of Economic Entomology.

[CR9] Envirologix Product. (2010). Pamphlet for EP-006- Imidacloprid Quantiplate Kit, Portland, ME. http://www.envirologix.com/library/ep006insert.pdf accessed 5/14/2012.

[CR10] EPA. (2008). Problem formulation for imidacloprid environmental fate and ecological risk assessment, US EPA, Washington DC http://www.regulations.gov/%23!documentDetail;D=EPA,HQ-OPP-2009-0081-0108 (accessed 2 Nov 2011).

[CR11] Fossen, M. (2006). Environmental fate of imidacloprid. Environmental Monitoring, California Department of Pesticide Regulation, April. This report can be accessed on line at http://www.cdpr.ca.gov/docs/emon/pubs/fatememo/Imidclprdfate2.pdf 7/8/12.

[CR12] Girolami V, Mazzon L, Squartini A, Mori N, Marzaro M, Di Bernardo A, Greatti M, Giorio C, Tapparo A (2009). Translocation of neonicotinoid insecticides from coated seeds to seedling guttation drops: a novel way of intoxication for bees. Journal of Economic Entomology.

[CR13] Gould JL, Gould CG (1995). The honey bee.

[CR14] Halm MP, Rortais A, Arnold G, Taséi JN, Rault S (2006). New risk assessment approach for systemic insecticides: the case of honey bees and imidacloprid (Gaucho). Environmental Science & Technology.

[CR15] Iwasa T, Motoyama N, Ambrose JT, Roe RM (2004). Mechanism for the differential toxicity of neonicotinoid insecticides in the honey bee, *Apis mellifera*. Crop Protection.

[CR16] Jeschke P, Nauen R, Schindler M, Elbert A (2011). Overview of the status and global strategy for neonicotinoids. Journal of Agricultural and Food Chemistry.

[CR17] Kreutzweiser DP, Thompson DG, Scarr TA (2009). Imidacloprid in leaves from systemically treated trees may inhibit litter breakdown by non-target invertebrates. Ecotoxicology and Environmental Safety.

[CR18] Krupke CH, Hunt GJ, Eitzer BD, Andino G, Given K (2012). Multiple routes of pesticide exposure for honey bees living near agricultural fields. PloS One.

[CR19] Kuhnholz S, Seeley TD (1997). The control of water collection in honey bee colonies. Behavioral Ecology and Sociobiology.

[CR20] Miles Inc. (1993). Imidacloprid: pesticide leaching potential model. Report No. 105008. This reference is within CDPR California Department of Pesticide Regulation http://www.cdpr.ca.gov/docs/emon/pubs/fatememo/imid.pdf accessed 5/8/2012.

[CR21] Mullin CA, Frazier M, Frazier JL, Ashcroft S, Simonds R, van Engelsdorp D, Pettis JS (2010). High levels of miticides and agrochemicals in North American apiaries: implications for honey bee health. PloS One.

[CR22] Nauen R, Ebbinghaus-Kintscher U, Schmuck R (2001). Toxicity and nicotinic acetylcholine receptor interaction of imidacloprid and its metabolites in *Apis mellifera* (Hymentoptera: Apidae). Pest Management Science.

[CR23] Pfeuffer, R. J., & Matson, F. (2001). Pesticide surface water quality report, March 2001 sampling event. [Online]. Available at http://www.sfwmd.gov/curre/pest/P0103rpt.pdf. (Verified 17 January 2006). South Florida Water Management District, West Palm Beach, FL. (Could not reaccess 3/19/12).

[CR24] Phillips, P. J., & Bode, R. W. (2002). Concentrations of pesticides and pesticide degradates in the Croton River watershed in southeastern New York, July-September 2000. [Online]. Available at http://ny.water.usgs.gov/pubs/wri/wri024063/wrir02-4063.pdf. (Verified 12 January 2006). USGS-NY, Troy, NY. (Could not reaccess on 3/19 2012).

[CR25] Romeh AA (2010). Phytoremediation of water and soil contaminated with imidacloprid pesticide by *Plantago major*, L. International Journal of Phytoremediatio.

[CR26] Rouchaud J, Thirion A, Wauters A, Van de Steene F, Benoit F, Ceustermans N, Gillet J, Marchand S, Vanparys L (1996). Effects of fertilizer on insecticides adsorption and biodegradation in crop soils. Archives of Environmental Contamination and Toxicology.

[CR27] Sarkar MA, Biswas PK, Roy S, Kole RK, Chowdhury A (1999). Effect of pH and type of formulation on the persistence of imidacloprid in water. Bulletin of Environmental Contamination and Toxicology.

[CR28] Schmuck R, Schoning R, Stork A, Schramel O (2001). Risk posed to honey bee (*Apis mellifera* L. Hymenoptera) by an imidacloprid seed dressing of sunflowers. Pest Management Science.

[CR29] Scholz, K., & Spiteller, M. (1992). Influence of groundcover on the degradation of 14C imidacloprid in soil. Brighton Crop protection Conference – Pest and Diseases. 883–888.

[CR30] Starner K, Goh KS (2012). Detections of the neonicotinoid insecticide imidacloprid in surface waters of three agricultural regions of California, USA, 2010–2011. Bulletin of Environmental Contamination and Toxicology.

[CR31] Suchail S, Guez D, Belzunces LP (2001). Discrepancy between acute and chronic toxicity induced by imidacloprid and its metabolites in *Apis mellifera*. Environmental Toxicology and Chemistry.

[CR32] Suchail S, Debrauwer L, Belzunces L (2004). Metabolism of imidacloprid in Apis mellifera. Pest Management Science.

[CR33] Sur R, Stork A (2003). Uptake, translocation and metabolism of imidacloprid in plants. Bulletin of Insectology.

[CR34] Tapparo A, Giorio C, Marzaro M, Marton D, Soldà L, Girolami V (2011). Rapid analysis of neonicotinoid insecticides in guttation drops of corn seedlings obtained from coated seeds. Journal of Environmental Monitoring.

[CR35] van Dijk, T. (2010). Effects of neonicotinoid pesticide pollution of Dutch surface water on non‐target species abundance. Final Thesis, Student number: 0444448, Sustainable Development, Track Land use, Environment and Biodiversity (SD: LEB), Utrecht University, Netherlands.

[CR36] Wamhoff H, Schneider V (1999). Photodegradation of imidacloprid. Journal of Agricultural and Food Chemistry.

